# 4-Hy­droxy-*N*,*N*-diiso­propyl­tryptammonium hydro­fumarate

**DOI:** 10.1107/S2414314625005644

**Published:** 2025-06-27

**Authors:** Marilyn Naeem, Andrew R. Chadeayne, James A. Golen, David R. Manke

**Affiliations:** a University of Massachusetts Dartmouth 285 Old Westport Road North Dartmouth MA 02747 USA; b CaaMTech, Inc., 58 Sunset Way, Suite 209, Issaquah, WA 98027, USA; University of Aberdeen, United Kingdom

**Keywords:** crystal structure, tryptamines, indoles, hydrogen bonding

## Abstract

The solid-state structure of the title salt, C_16_H_25_N_2_O^+^·C_4_H_3_O_4_^−^ {systematic name: [2-(4-hy­droxy-1*H*-indol-3-yl)eth­yl]bis­(propan-2-yl)aza­nium (2*E*)-3-carb­oxy­prop-2-enoate}, is reported. In the extended structure, the hydro­fumarate anions form linear chains propagating in the [100] direction through O—H⋯O hydrogen bonds that combine with the tryptammonium cations to generate a three-dimensional network linked by O—H⋯O and N—H⋯O hydrogen bonds.

## Abstract 2

The solid-state structure of the hydro­fumarate salt of the synthetic psychedelic 4-hy­droxy-*N*,*N*-diiso­propyl­tryptamine (4-HO-DiPT) is reported.

## Graphical abstract

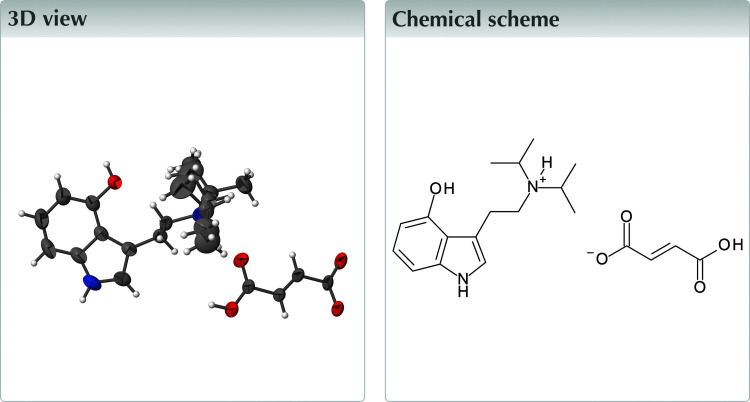

## Structure description

4-Hy­droxy-*N*,*N*-diiso­propyl­tryptamine (4-HO-DiPT, C_16_H_24_N_2_O) is a synthetic structural analog of serotonin (5-hy­droxy­tryptamine), which differs by moving the hy­droxy moiety from the 5- to the 4- position of the indole unit, and di-alkyl­ating its primary amine with two isopropyl groups. Serotonin analogs have been the subject of fascination for millenia because they alter human perception and consciousness when ingested (George *et al.*, 2022 ▸), effects now attributed to the 5-HT_2 A_ receptor (Halberstadt & Geyer, 2011 ▸). Psilocybin (4-phosphor­yloxy-*N*,*N*-di­methyl­tryptamine) and psilocin (4-hy­droxy-*N*,*N*-di­methyl­tryptamine) are widely known examples, both natural products found in at least 200 species of so-called ‘magic’ mushrooms (Nichols, 2020 ▸). Other naturally occurring serotonin analogs (*e.g.* 5-MeO-DMT, DMT, bufotenine) have been identified in plants and animals (Araújo *et al.*, 2015 ▸). Different structural analogs of serotonin produce varied perceptual and biological effects in humans.

Recently, several studies have indicated that 5-HT_2 A_ agonists hold tremendous potential to treat the most harmful and intra­ctable mental health conditions including depression, anxiety and post-traumatic stress disorder (PTSD). Psilocybin and other psilocin prodrugs are currently being developed as a treatment for treatment-resistant depression (TRD), anorexia and PTSD (Goodwin *et al.*, 2022 ▸). 5-Meth­oxy-*N*,*N*-di­methyl­tryptamine (5-MeO-DMT) is also in Phase 2 trials as treatment for TRD (Reckweg *et al.*, 2023 ▸). A prodrug of the title compound is in Phase 2 clinical trials for the treatment of post partem depression and adjustment disorder (NIH, 2025 ▸).

Alongside these clinical studies, others have sought to elucidate a structure–activity relationship (SAR), by understanding how the structural differences across serotonin analogs correlate with their pharmacoogical and, ultimately, clinical differences in human subjects. 4-Hy­droxy-*N*,*N*-diiso­propyl­tryptamine (4-HO-DiPT) was first synthesized in 1977 as its hydro­chloride salt (Repke *et al.*, 1977 ▸). This sterically bulky analogue of psilocin is more selective toward serotonin receptors, binding to three of the possible 14 receptors, while psilocin and less bulky analogues often bind to ten (Glatfelter *et al.*, 2023 ▸). This synthetic variant of psilocin has been noted for its fast onset, brevity and intensity of action (Shulgin & Shulgin, 2016 ▸).

In 2022, the US Drug Enforcement Agency proposed reclassifying 4-HO-DiPT to Schedule I of the Controlled Substance Act; the proposal was withdrawn due to strong public response (US DEA, January 14 and July 6, 2022*a* ▸,*b* ▸). Alongside this proposed rescheduling of 4-HO-DiPT, Reunion Neuroscience (Toronto, Canada) initiated clinical trials of a ‘hemi-ester’ prodrug of the same, *i.e.* 4-glutarato-*N*,*N*-diiso­propyl­tryptamine. We have published ethanol and methanol solvates of this prodrug, which are the first two crystal structures of this compound, and also the first structures of any *N*,*N*-diiso­propyl­tryptamine (Naeem *et al.*, 2022 ▸). The propensity of this prodrug to form solvates in the solid state could potentially explain the difficulties removing impurities described in the later report on the synthesis and activity of this compound (Bryson *et al.*, 2024 ▸). While we have reported the first crystal structures of these prodrugs, the structure of the active metabolite has been absent. Herein, we report the first crystal structure of 4-hy­droxy-*N*,*N*-diiso­propyl­tryptamine as its hydro­fumarate salt.

**Figure 1:**
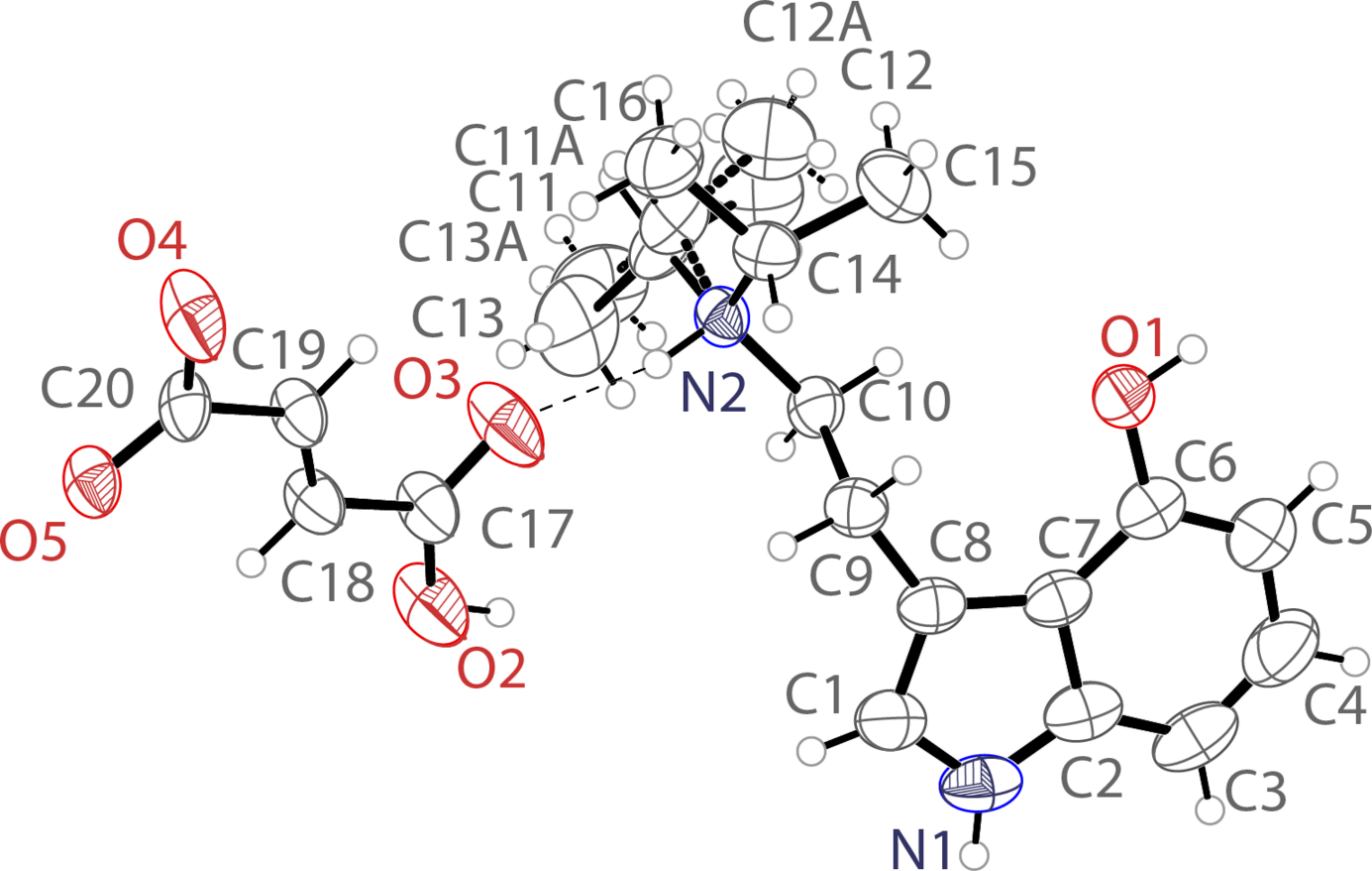
The mol­ecular structure of the title compound showing the atomic labeling. Displacement ellipsoids are drawn at the 50% probability level. Dashed bonds indicate a disordered component in the structure. Hydrogen bonds are shown as dashed lines.

**Figure 2:**
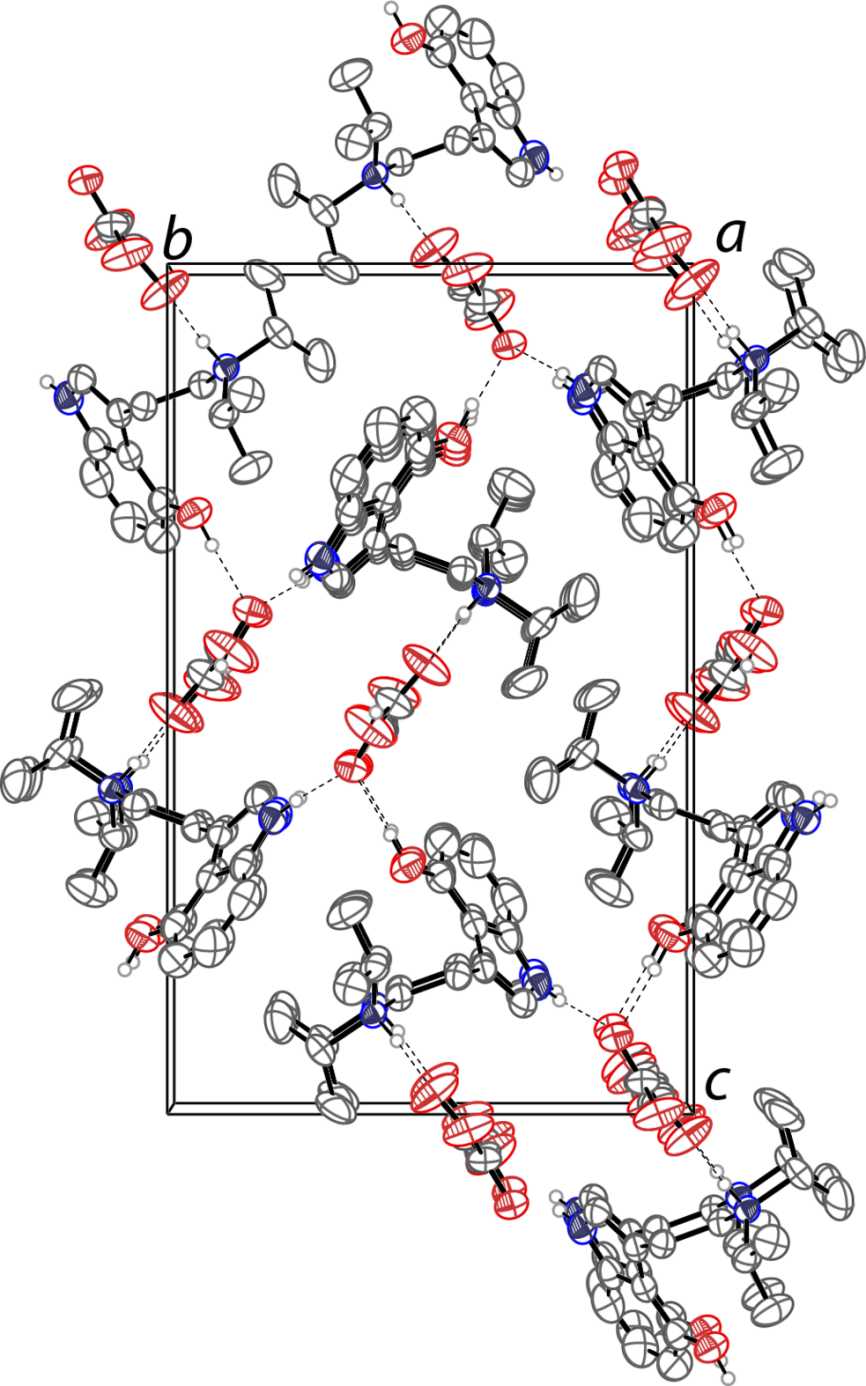
The crystal packing of the title compound viewed along the *a* axis. The hydrogen bonds are shown as dashed lines. Hydrogen atoms not involved in hydrogen bonds are omitted for clarity. Only one component of the disordered isopropyl groups are shown.

The mol­ecular structure of the title compound is shown in Fig. 1 ▸. The asymmetric unit contains one 4-hy­droxy-*N*,*N*-diiso­propyl­tryptammonium (C_16_H_25_N_2_O^+^) cation and one hydro­fumarate (C_4_H_3_O_4_^−^) anion. The indole ring of the cation is near planar with a r.m.s. deviation from planarity of 0.005 Å for the non-hydrogen atoms. The hydro­fumarate anion is slightly less planar with a r.m.s. deviation from planarity of 0.081 Å for the non-hydrogen atoms. The ethyl­amino arm of the tryptamine is turned away from the indole plane with a C7—C8—C9—C10 torsion angle of 75.9 (3)° and the C8—C9—C10—N2 grouping has an *anti* conformation [torsion angle = 170.4 (2)°]. One of the isopropyl groups (C11–C13) is disordered over two orientations in a 0.51 (4):0.49 (4) ratio. In the extended structure, the hydro­fumarate anions form linear chains along [100] through O—H⋯O hydrogen bonds (Table 1 ▸, Fig. 2 ▸). These chains are connected into a three-dimensional network by O—H⋯O and N—H⋯O hydrogen bonds with the tryptammonium cations.

In addition to the structure reported here, there are eight other hydro­fumarate salts of tryptamines reported including those of *N*-ethyl-*N*-*n*-propyl­tryptamine (Cambridge Structural Database refcode GUPBOL; Chadeayne *et al.*, 2020*c* ▸) and *N*-allyl-*N*-methyl­tryptamine (GUPBUR: Chadeayne *et al.*, 2020*c* ▸), 4-acet­oxy-*N*,*N*-di­methyl­tryptamine (HOCJUH; Chadeayne *et al.*, 2019*a* ▸), 4-acet­oxy-*N*-ethyl-*N*-methyl­tryptamine (OJIQIK; Pham *et al.*, 2021*a* ▸) and 4-acet­oxy-*N*-allyl-*N*-methyl­tryptamine (OJIQUQ; Pham *et al.*, 2021*a* ▸), *N*-methyl-*N*-iso­propyl­tryptamine (RONSOF; Chadeayne *et al.*, 2019*c* ▸) and 4-hy­droxy-*N*-methyl-*N*-iso­propyl­tryptamine (RONSUL; Chadeayne *et al.*, 2019*c* ▸), and 4-propion­oxy-*N*,*N*-di­methyl­tryptamine (Glatfelter *et al.*, 2023 ▸). The other tryptamine structures reported with fumaric acid based counter-ions are nine (2:1) tryptamine:fumarate salts including those of the anti-cancer drug panobinostat (MIMMAA: Kenguva *et al.*, 2023 ▸), the mushroom natural product norpsilocin (MULXEZ: Chadeayne *et al.*, 2020*b* ▸), 5-meth­oxy-*N*,*N*-di­allyl­tryptamine (OPUDEL: Pham *et al.*, 2021*c* ▸), 5-meth­oxy-*N*,*N*-di-*n*-propyl­tryptamine (OQIGON: Pham *et al.*, 2021*d* ▸), 4-hyd­oxy-*N*-methyl-*N*-iso­propyl­tryptamine (TUFQAP: Chadeayne *et al.*, 2020*a* ▸), 5-meth­oxy-2-methyl-*N*,*N*-di­methyl­tryptamine (ULUTED: Pham *et al.*, 2021*b* ▸), 4-hy­droxy-*N*,*N*-di-*n*-propyl­tryptamine (WUCGAF: Chadeayne *et al.*, 2019*d* ▸), psilacetin (XOFDOO: Chadeayne *et al.*, 2019*b* ▸), *N*-cyclo­hexyl­tryptamine (YITWIL: Naeem *et al.*, 2023 ▸), and two (2:1:1) tryptamine:fumarate:fumaric acid complexes, being those of 4-acet­oxy-*N*-ethyl-*N*-*n*-propyl­tryptamine (BIYKED: Pham *et al.*, 2023 ▸) and 4-acet­oxy-*N*,*N*-di­allyl­tryptamine (OJIQUW: Pham *et al.*, 2021*a* ▸). There are also two structures of the 4-HO-DiPT prodrug 4-glutarato-*N*,*N*-diiso­propyl­tryptamine reported (TEKWOZ, TEKWUF: Naeem *et al.*, 2022 ▸).

## Synthesis and crystallization

Slow evaporation of a methanol/water solution of a commercial sample of ‘4-HO-DiPT fumarate’ (Chem Logix) resulted in the formation of colorless blocks of the title compound suitable for X-ray analysis.

## Refinement

Crystal data, data collection and structure refinement details are summarized in Table 2 ▸. The C11–C13 isopropyl group is disordered over two orientations in a 0.51 (4):0.49 (4) ratio. The disorder model was restrained with N—C distances of 1.50 (1) Å, as well as SADI C—C distance restraints, DELU rigid body restraints, and ISOR isotropic restraints. The structure was refined as an inversion twin.

## Supplementary Material

Crystal structure: contains datablock(s) I. DOI: 10.1107/S2414314625005644/hb4526sup1.cif

Structure factors: contains datablock(s) I. DOI: 10.1107/S2414314625005644/hb4526Isup2.hkl

Supporting information file. DOI: 10.1107/S2414314625005644/hb4526Isup3.cml

CCDC reference: 2466286

Additional supporting information:  crystallographic information; 3D view; checkCIF report

## Figures and Tables

**Table 1:** Hydrogen-bond geometry (Å, °)

*D*—H⋯*A*	*D*—H	H⋯*A*	*D*⋯*A*	*D*—H⋯*A*
O2—H2*A*⋯O4^i^	0.91 (1)	1.54 (2)	2.441 (3)	173 (6)
N1—H1*A*⋯O5^ii^	0.87 (1)	2.13 (2)	2.989 (3)	170 (4)
N2—H2⋯O3	0.90 (1)	1.88 (1)	2.756 (3)	165 (2)
O1—H1⋯O5^iii^	0.89 (1)	1.89 (1)	2.784 (3)	177 (4)

Symmetry codes: (i) 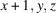; (ii) 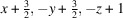; (iii) 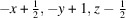.

**Table 2:** Experimental details

Crystal data	
Chemical formula	C_16_H_25_N_2_O^+^·C_4_H_3_O_4_^−^
*M*_r_	376.44
Crystal system, space group	Orthorhombic, *P*2_1_2_1_2_1_
Temperature (K)	300
*a*, *b*, *c* (Å)	7.9541 (3), 12.5763 (5), 20.3351 (6)
*V* (Å^3^)	2034.18 (13)
*Z*	4
Radiation type	Mo *K*α
μ (mm^−1^)	0.09
Crystal size (mm)	0.38 × 0.24 × 0.12

Data collection	
Diffractometer	Bruker D8 Venture CMOS
Absorption correction	Multi-scan (*SADABS*; Krause *et al.*, 2015 ▸)
*T*_min_, *T*_max_	0.714, 0.745
No. of measured, independent and observed [*I* > 2σ(*I*)] reflections	23480, 3864, 3485
*R*_int_	0.034
(sin θ/λ)_max_ (Å^−1^)	0.611

Refinement	
*R*[*F*^2^ > 2σ(*F*^2^)], *wR*(*F*^2^), *S*	0.039, 0.095, 1.08
No. of reflections	3864
No. of parameters	295
No. of restraints	54
H-atom treatment	H atoms treated by a mixture of independent and constrained refinement
Δρ_max_, Δρ_min_ (e Å^−3^)	0.14, −0.12
Absolute structure	Refined as an inversion twin
Absolute structure parameter	0.6 (15)

Computer programs: *APEX3* and *SAINT* (Bruker, 2018 ▸), *SHELXT2014* (Sheldrick, 2015*a* ▸), *SHELXL2018* (Sheldrick, 2015*b* ▸), *OLEX2* (Dolomanov *et al.*, 2009 ▸) and *publCIF* (Westrip, 2010 ▸).

## full crystallographic data

### [2-(4-Hydroxy-1H-indol-3-yl)ethyl]bis(propan-2-yl)azanium (2E)-3-carboxyprop-2-enoate Crystal data

**Table:**

C_16_H_25_N_2_O^+^·C_4_H_3_O_4_^−^	*D*_x_ = 1.229 Mg m^−^^3^
*M**_r_* = 376.44	Mo *K*α radiation, λ = 0.71073 Å
Orthorhombic, *P*2_1_2_1_2_1_	Cell parameters from 9289 reflections
*a* = 7.9541 (3) Å	θ = 2.8–25.6°
*b* = 12.5763 (5) Å	µ = 0.09 mm^−^^1^
*c* = 20.3351 (6) Å	*T* = 300 K
*V* = 2034.18 (13) Å^3^	Block, colourless
*Z* = 4	0.38 × 0.24 × 0.12 mm
*F*(000) = 808	

### [2-(4-Hydroxy-1H-indol-3-yl)ethyl]bis(propan-2-yl)azanium (2E)-3-carboxyprop-2-enoate Data collection

**Table:**

Bruker D8 Venture CMOS diffractometer	3485 reflections with *I* > 2σ(*I*)
φ and ω scans	*R*_int_ = 0.034
Absorption correction: multi-scan (SADABS; Krause *et al.*, 2015)	θ_max_ = 25.7°, θ_min_ = 2.6°
*T*_min_ = 0.714, *T*_max_ = 0.745	*h* = −9→9
23480 measured reflections	*k* = −15→15
3864 independent reflections	*l* = −18→24

### [2-(4-Hydroxy-1H-indol-3-yl)ethyl]bis(propan-2-yl)azanium (2E)-3-carboxyprop-2-enoate Refinement

**Table:**

Refinement on *F*^2^	Hydrogen site location: mixed
Least-squares matrix: full	H atoms treated by a mixture of independent and constrained refinement
*R*[*F*^2^ > 2σ(*F*^2^)] = 0.039	*w* = 1/[σ^2^(*F*_o_^2^) + (0.0408*P*)^2^ + 0.3705*P*] where *P* = (*F*_o_^2^ + 2*F*_c_^2^)/3
*wR*(*F*^2^) = 0.095	(Δ/σ)_max_ = 0.001
*S* = 1.08	Δρ_max_ = 0.14 e Å^−^^3^
3864 reflections	Δρ_min_ = −0.12 e Å^−^^3^
295 parameters	Absolute structure: Refined as an inversion twin
54 restraints	Absolute structure parameter: 0.6 (15)
Primary atom site location: dual	

### [2-(4-Hydroxy-1H-indol-3-yl)ethyl]bis(propan-2-yl)azanium (2E)-3-carboxyprop-2-enoate Special details

**Table:**

Geometry. All esds (except the esd in the dihedral angle between two l.s. planes) are estimated using the full covariance matrix. The cell esds are taken into account individually in the estimation of esds in distances, angles and torsion angles; correlations between esds in cell parameters are only used when they are defined by crystal symmetry. An approximate (isotropic) treatment of cell esds is used for estimating esds involving l.s. planes.
Refinement. Atoms H1, H1*A*, H2, and H2*A* were found from difference-Fourier maps and allowed to refine with restrained an N—H distance of 0.87 (1) Å and 1.20 *U*_eq_ of the parent indole nitrogen, an N—H distance 0.90 (1) Å and 1.20 *U*_eq_ of the parent ammonium nitrogen, and O—H distances of 0.90 (1) Å and 1.50 *U*_eq_ of parent oxygen atoms. All other hydrogen atoms were placed in calculated positions with appropriate riding parameters. Refined as a 2-component inversion twin.

### [2-(4-Hydroxy-1H-indol-3-yl)ethyl]bis(propan-2-yl)azanium (2E)-3-carboxyprop-2-enoate Fractional atomic coordinates and isotropic or equivalent isotropic displacement parameters (Å^2^)

**Table:**

	*x*	*y*	*z*	*U*_iso_*/*U*_eq_	Occ. (<1)
O1	0.7205 (3)	0.45612 (17)	0.21046 (10)	0.0586 (5)	
O2	0.3240 (2)	0.6148 (2)	0.54461 (13)	0.0875 (9)	
H2A	0.427 (3)	0.602 (4)	0.527 (3)	0.16 (2)*	
O3	0.2145 (2)	0.5076 (2)	0.47141 (12)	0.0839 (8)	
O4	−0.3942 (2)	0.5705 (2)	0.50563 (10)	0.0791 (8)	
O5	−0.2902 (2)	0.65068 (16)	0.59369 (8)	0.0486 (4)	
N1	0.9479 (3)	0.7045 (2)	0.35061 (13)	0.0586 (7)	
N2	0.3986 (3)	0.39208 (16)	0.38155 (10)	0.0406 (5)	
C1	0.7910 (4)	0.6762 (2)	0.37158 (14)	0.0531 (7)	
H1B	0.738117	0.703461	0.408711	0.064*	
C2	0.9858 (3)	0.6496 (2)	0.29497 (14)	0.0518 (7)	
C3	1.1308 (4)	0.6528 (3)	0.25608 (19)	0.0673 (9)	
H3	1.221446	0.696320	0.266611	0.081*	
C4	1.1325 (4)	0.5891 (3)	0.2022 (2)	0.0764 (10)	
H4	1.227306	0.589094	0.175423	0.092*	
C5	0.9968 (4)	0.5233 (3)	0.18541 (17)	0.0689 (9)	
H5	1.003159	0.481506	0.147773	0.083*	
C6	0.8555 (4)	0.5198 (2)	0.22348 (15)	0.0514 (7)	
C7	0.8470 (3)	0.58421 (19)	0.28019 (13)	0.0435 (6)	
C8	0.7230 (3)	0.60230 (19)	0.33038 (12)	0.0440 (6)	
C9	0.5517 (3)	0.5530 (2)	0.33740 (13)	0.0440 (6)	
H9A	0.495522	0.553065	0.295076	0.053*	
H9B	0.484818	0.595014	0.367676	0.053*	
C10	0.5650 (3)	0.4400 (2)	0.36269 (12)	0.0411 (5)	
H10A	0.616347	0.396122	0.328976	0.049*	
H10B	0.638543	0.439132	0.400747	0.049*	
C11	0.427 (2)	0.2918 (10)	0.4237 (8)	0.052 (3)	0.49 (4)
H11	0.318518	0.255912	0.428418	0.062*	0.49 (4)
C12	0.547 (3)	0.2139 (15)	0.3918 (12)	0.101 (5)	0.49 (4)
H12A	0.533434	0.145121	0.411493	0.151*	0.49 (4)
H12B	0.660493	0.237862	0.397948	0.151*	0.49 (4)
H12C	0.522964	0.209468	0.345594	0.151*	0.49 (4)
C13	0.488 (3)	0.320 (2)	0.4921 (8)	0.092 (5)	0.49 (4)
H13A	0.404651	0.362825	0.513684	0.138*	0.49 (4)
H13B	0.591273	0.358873	0.489015	0.138*	0.49 (4)
H13C	0.505785	0.255956	0.516831	0.138*	0.49 (4)
C11A	0.402 (2)	0.2809 (8)	0.4102 (8)	0.054 (3)	0.51 (4)
H11A	0.284595	0.259997	0.417814	0.065*	0.51 (4)
C12A	0.479 (3)	0.1967 (11)	0.3682 (11)	0.096 (5)	0.51 (4)
H12D	0.493440	0.132917	0.393531	0.144*	0.51 (4)
H12E	0.586697	0.220607	0.352648	0.144*	0.51 (4)
H12F	0.407206	0.182372	0.331322	0.144*	0.51 (4)
C13A	0.484 (3)	0.2892 (16)	0.4766 (11)	0.091 (4)	0.51 (4)
H13D	0.474229	0.222598	0.499337	0.137*	0.51 (4)
H13E	0.430600	0.344092	0.501814	0.137*	0.51 (4)
H13F	0.601223	0.306291	0.471114	0.137*	0.51 (4)
C14	0.2691 (4)	0.3954 (2)	0.32555 (13)	0.0512 (7)	
H14	0.250591	0.470741	0.315689	0.061*	
C15	0.3301 (5)	0.3449 (3)	0.26212 (17)	0.0861 (12)	
H15A	0.436477	0.375199	0.250051	0.129*	
H15B	0.249718	0.357998	0.227835	0.129*	
H15C	0.342555	0.269678	0.268344	0.129*	
C16	0.1007 (4)	0.3515 (3)	0.34731 (17)	0.0685 (8)	
H16A	0.069079	0.383492	0.388358	0.103*	
H16B	0.109015	0.275878	0.352725	0.103*	
H16C	0.017280	0.367423	0.314643	0.103*	
C17	0.2024 (3)	0.5696 (3)	0.51642 (13)	0.0529 (7)	
C18	0.0350 (3)	0.5989 (2)	0.54360 (14)	0.0507 (7)	
H18	0.030972	0.640540	0.581360	0.061*	
C19	−0.1049 (3)	0.5692 (2)	0.51722 (12)	0.0454 (6)	
H19	−0.099546	0.525415	0.480425	0.055*	
C20	−0.2747 (3)	0.6005 (2)	0.54185 (12)	0.0437 (6)	
H1A	1.015 (4)	0.749 (2)	0.3704 (16)	0.093 (13)*	
H2	0.355 (3)	0.4362 (17)	0.4119 (10)	0.040 (7)*	
H1	0.739 (5)	0.423 (3)	0.1724 (11)	0.082 (11)*	

### [2-(4-Hydroxy-1H-indol-3-yl)ethyl]bis(propan-2-yl)azanium (2E)-3-carboxyprop-2-enoate Atomic displacement parameters (Å^2^)

**Table:**

	*U*^11^	*U*^22^	*U*^33^	*U*^12^	*U*^13^	*U*^23^
O1	0.0515 (11)	0.0691 (13)	0.0553 (11)	−0.0176 (10)	0.0101 (9)	−0.0149 (10)
O2	0.0232 (10)	0.141 (2)	0.0983 (18)	−0.0018 (12)	0.0063 (10)	−0.0682 (17)
O3	0.0291 (10)	0.131 (2)	0.0915 (16)	0.0101 (11)	0.0009 (10)	−0.0680 (16)
O4	0.0235 (9)	0.152 (2)	0.0618 (12)	0.0044 (12)	0.0002 (8)	−0.0417 (14)
O5	0.0306 (9)	0.0669 (11)	0.0482 (10)	0.0073 (9)	0.0045 (8)	−0.0080 (9)
N1	0.0605 (16)	0.0487 (13)	0.0666 (16)	−0.0138 (12)	−0.0207 (13)	0.0029 (12)
N2	0.0406 (11)	0.0381 (10)	0.0430 (10)	0.0058 (9)	0.0081 (9)	−0.0021 (9)
C1	0.0628 (18)	0.0453 (14)	0.0510 (15)	−0.0064 (14)	−0.0091 (14)	0.0023 (11)
C2	0.0486 (16)	0.0426 (14)	0.0641 (17)	−0.0055 (12)	−0.0159 (13)	0.0105 (13)
C3	0.0415 (17)	0.0646 (19)	0.096 (3)	−0.0155 (15)	−0.0073 (16)	0.0174 (19)
C4	0.054 (2)	0.077 (2)	0.098 (3)	−0.0126 (17)	0.0181 (18)	0.006 (2)
C5	0.060 (2)	0.073 (2)	0.073 (2)	−0.0121 (16)	0.0139 (16)	−0.0047 (17)
C6	0.0463 (16)	0.0497 (15)	0.0580 (16)	−0.0083 (12)	−0.0004 (12)	0.0037 (13)
C7	0.0413 (14)	0.0375 (13)	0.0517 (14)	−0.0033 (10)	−0.0075 (11)	0.0075 (11)
C8	0.0467 (14)	0.0356 (12)	0.0498 (13)	−0.0013 (11)	−0.0074 (11)	0.0039 (10)
C9	0.0431 (14)	0.0412 (13)	0.0478 (13)	0.0012 (11)	−0.0017 (11)	−0.0015 (11)
C10	0.0356 (13)	0.0432 (12)	0.0447 (12)	0.0030 (11)	0.0053 (10)	−0.0013 (11)
C11	0.048 (5)	0.049 (4)	0.058 (5)	−0.003 (3)	0.002 (4)	0.019 (4)
C12	0.129 (10)	0.059 (6)	0.114 (9)	0.032 (7)	0.013 (7)	0.012 (5)
C13	0.115 (9)	0.098 (10)	0.062 (6)	0.019 (8)	−0.018 (5)	0.033 (5)
C11A	0.045 (5)	0.053 (4)	0.064 (5)	−0.001 (3)	0.002 (4)	0.010 (3)
C12A	0.126 (10)	0.044 (5)	0.117 (8)	0.028 (6)	0.018 (7)	0.006 (5)
C13A	0.111 (8)	0.072 (7)	0.091 (8)	−0.002 (6)	−0.025 (7)	0.025 (6)
C14	0.0531 (16)	0.0514 (14)	0.0490 (14)	−0.0098 (13)	−0.0036 (12)	−0.0052 (12)
C15	0.087 (3)	0.107 (3)	0.063 (2)	−0.029 (2)	0.0059 (18)	−0.033 (2)
C16	0.0541 (17)	0.077 (2)	0.074 (2)	−0.0149 (17)	−0.0052 (16)	0.0004 (17)
C17	0.0242 (12)	0.0787 (19)	0.0558 (15)	0.0062 (13)	0.0005 (11)	−0.0235 (15)
C18	0.0270 (12)	0.0701 (18)	0.0550 (14)	0.0015 (12)	0.0032 (11)	−0.0246 (14)
C19	0.0284 (12)	0.0645 (17)	0.0434 (12)	0.0076 (12)	0.0016 (10)	−0.0127 (12)
C20	0.0258 (11)	0.0672 (16)	0.0382 (12)	0.0038 (11)	0.0038 (10)	−0.0017 (12)

### [2-(4-Hydroxy-1H-indol-3-yl)ethyl]bis(propan-2-yl)azanium (2E)-3-carboxyprop-2-enoate Geometric parameters (Å, º)

**Table:**

O1—C6	1.365 (3)	C11—H11	0.9800
O1—H1	0.891 (13)	C11—C12	1.512 (9)
O2—H2A	0.908 (14)	C11—C13	1.512 (9)
O2—C17	1.260 (3)	C12—H12A	0.9600
O3—C17	1.206 (3)	C12—H12B	0.9600
O4—C20	1.260 (3)	C12—H12C	0.9600
O5—C20	1.235 (3)	C13—H13A	0.9600
N1—C1	1.366 (4)	C13—H13B	0.9600
N1—C2	1.359 (4)	C13—H13C	0.9600
N1—H1A	0.870 (13)	C11A—H11A	0.9800
N2—C10	1.504 (3)	C11A—C12A	1.494 (9)
N2—C11	1.543 (9)	C11A—C13A	1.506 (9)
N2—C11A	1.515 (10)	C12A—H12D	0.9600
N2—C14	1.536 (3)	C12A—H12E	0.9600
N2—H2	0.899 (13)	C12A—H12F	0.9600
C1—H1B	0.9300	C13A—H13D	0.9600
C1—C8	1.363 (4)	C13A—H13E	0.9600
C2—C3	1.400 (4)	C13A—H13F	0.9600
C2—C7	1.409 (4)	C14—H14	0.9800
C3—H3	0.9300	C14—C15	1.517 (4)
C3—C4	1.357 (5)	C14—C16	1.515 (4)
C4—H4	0.9300	C15—H15A	0.9600
C4—C5	1.402 (5)	C15—H15B	0.9600
C5—H5	0.9300	C15—H15C	0.9600
C5—C6	1.366 (4)	C16—H16A	0.9600
C6—C7	1.411 (4)	C16—H16B	0.9600
C7—C8	1.437 (4)	C16—H16C	0.9600
C8—C9	1.503 (4)	C17—C18	1.488 (3)
C9—H9A	0.9700	C18—H18	0.9300
C9—H9B	0.9700	C18—C19	1.290 (3)
C9—C10	1.516 (4)	C19—H19	0.9300
C10—H10A	0.9700	C19—C20	1.493 (3)
C10—H10B	0.9700		
			
C6—O1—H1	108 (2)	C11—C12—H12C	109.5
C17—O2—H2A	116 (4)	H12A—C12—H12B	109.5
C1—N1—H1A	126 (3)	H12A—C12—H12C	109.5
C2—N1—C1	109.2 (2)	H12B—C12—H12C	109.5
C2—N1—H1A	125 (3)	C11—C13—H13A	109.5
C10—N2—C11	109.8 (7)	C11—C13—H13B	109.5
C10—N2—C11A	117.0 (6)	C11—C13—H13C	109.5
C10—N2—C14	112.97 (18)	H13A—C13—H13B	109.5
C10—N2—H2	105.3 (16)	H13A—C13—H13C	109.5
C11—N2—H2	100.3 (17)	H13B—C13—H13C	109.5
C11A—N2—C14	108.7 (8)	N2—C11A—H11A	107.0
C11A—N2—H2	108.2 (17)	C12A—C11A—N2	116.2 (10)
C14—N2—C11	122.2 (8)	C12A—C11A—H11A	107.0
C14—N2—H2	103.7 (16)	C12A—C11A—C13A	112.5 (11)
N1—C1—H1B	124.8	C13A—C11A—N2	106.7 (11)
C8—C1—N1	110.4 (3)	C13A—C11A—H11A	107.0
C8—C1—H1B	124.8	C11A—C12A—H12D	109.5
N1—C2—C3	129.7 (3)	C11A—C12A—H12E	109.5
N1—C2—C7	107.5 (3)	C11A—C12A—H12F	109.5
C3—C2—C7	122.8 (3)	H12D—C12A—H12E	109.5
C2—C3—H3	121.7	H12D—C12A—H12F	109.5
C4—C3—C2	116.6 (3)	H12E—C12A—H12F	109.5
C4—C3—H3	121.7	C11A—C13A—H13D	109.5
C3—C4—H4	118.8	C11A—C13A—H13E	109.5
C3—C4—C5	122.5 (3)	C11A—C13A—H13F	109.5
C5—C4—H4	118.8	H13D—C13A—H13E	109.5
C4—C5—H5	119.5	H13D—C13A—H13F	109.5
C6—C5—C4	121.0 (3)	H13E—C13A—H13F	109.5
C6—C5—H5	119.5	N2—C14—H14	106.2
O1—C6—C5	123.8 (3)	C15—C14—N2	113.9 (3)
O1—C6—C7	117.2 (2)	C15—C14—H14	106.2
C5—C6—C7	118.9 (3)	C16—C14—N2	111.5 (2)
C2—C7—C6	118.2 (2)	C16—C14—H14	106.2
C2—C7—C8	107.1 (2)	C16—C14—C15	112.3 (3)
C6—C7—C8	134.8 (2)	C14—C15—H15A	109.5
C1—C8—C7	105.8 (2)	C14—C15—H15B	109.5
C1—C8—C9	125.6 (3)	C14—C15—H15C	109.5
C7—C8—C9	128.6 (2)	H15A—C15—H15B	109.5
C8—C9—H9A	109.5	H15A—C15—H15C	109.5
C8—C9—H9B	109.5	H15B—C15—H15C	109.5
C8—C9—C10	110.8 (2)	C14—C16—H16A	109.5
H9A—C9—H9B	108.1	C14—C16—H16B	109.5
C10—C9—H9A	109.5	C14—C16—H16C	109.5
C10—C9—H9B	109.5	H16A—C16—H16B	109.5
N2—C10—C9	113.63 (19)	H16A—C16—H16C	109.5
N2—C10—H10A	108.8	H16B—C16—H16C	109.5
N2—C10—H10B	108.8	O2—C17—C18	114.0 (2)
C9—C10—H10A	108.8	O3—C17—O2	125.1 (2)
C9—C10—H10B	108.8	O3—C17—C18	120.9 (2)
H10A—C10—H10B	107.7	C17—C18—H18	118.5
N2—C11—H11	107.4	C19—C18—C17	123.0 (2)
C12—C11—N2	112.5 (9)	C19—C18—H18	118.5
C12—C11—H11	107.4	C18—C19—H19	117.8
C13—C11—N2	111.5 (10)	C18—C19—C20	124.3 (2)
C13—C11—H11	107.4	C20—C19—H19	117.8
C13—C11—C12	110.2 (11)	O4—C20—C19	114.1 (2)
C11—C12—H12A	109.5	O5—C20—O4	125.2 (2)
C11—C12—H12B	109.5	O5—C20—C19	120.8 (2)
			
O1—C6—C7—C2	179.3 (2)	C6—C7—C8—C9	0.2 (5)
O1—C6—C7—C8	−1.4 (4)	C7—C2—C3—C4	0.2 (4)
O2—C17—C18—C19	173.0 (3)	C7—C8—C9—C10	75.9 (3)
O3—C17—C18—C19	−7.1 (5)	C8—C9—C10—N2	170.4 (2)
N1—C1—C8—C7	−0.3 (3)	C10—N2—C11—C12	−52.0 (12)
N1—C1—C8—C9	−179.5 (2)	C10—N2—C11—C13	72.4 (13)
N1—C2—C3—C4	−179.3 (3)	C10—N2—C11A—C12A	−57.9 (12)
N1—C2—C7—C6	179.2 (2)	C10—N2—C11A—C13A	68.4 (13)
N1—C2—C7—C8	−0.3 (3)	C10—N2—C14—C15	54.5 (3)
C1—N1—C2—C3	179.6 (3)	C10—N2—C14—C16	−177.2 (2)
C1—N1—C2—C7	0.1 (3)	C11—N2—C10—C9	−164.4 (7)
C1—C8—C9—C10	−105.0 (3)	C11—N2—C14—C15	−80.1 (6)
C2—N1—C1—C8	0.1 (3)	C11—N2—C14—C16	48.1 (6)
C2—C3—C4—C5	0.3 (5)	C11A—N2—C10—C9	−177.3 (8)
C2—C7—C8—C1	0.3 (3)	C11A—N2—C14—C15	−77.0 (6)
C2—C7—C8—C9	179.6 (2)	C11A—N2—C14—C16	51.2 (6)
C3—C2—C7—C6	−0.3 (4)	C14—N2—C10—C9	55.3 (3)
C3—C2—C7—C8	−179.8 (3)	C14—N2—C11—C12	83.9 (12)
C3—C4—C5—C6	−0.6 (6)	C14—N2—C11—C13	−151.7 (11)
C4—C5—C6—O1	−178.8 (3)	C14—N2—C11A—C12A	71.5 (13)
C4—C5—C6—C7	0.5 (5)	C14—N2—C11A—C13A	−162.2 (12)
C5—C6—C7—C2	0.0 (4)	C17—C18—C19—C20	−177.7 (3)
C5—C6—C7—C8	179.3 (3)	C18—C19—C20—O4	174.1 (3)
C6—C7—C8—C1	−179.1 (3)	C18—C19—C20—O5	−6.3 (4)

### [2-(4-Hydroxy-1H-indol-3-yl)ethyl]bis(propan-2-yl)azanium (2E)-3-carboxyprop-2-enoate Hydrogen-bond geometry (Å, º)

**Table:**

*D*—H···*A*	*D*—H	H···*A*	*D*···*A*	*D*—H···*A*
O2—H2*A*···O4^i^	0.91 (1)	1.54 (2)	2.441 (3)	173 (6)
N1—H1*A*···O5^ii^	0.87 (1)	2.13 (2)	2.989 (3)	170 (4)
N2—H2···O3	0.90 (1)	1.88 (1)	2.756 (3)	165 (2)
O1—H1···O5^iii^	0.89 (1)	1.89 (1)	2.784 (3)	177 (4)

Symmetry codes: (i) *x*+1, *y*, *z*; (ii) *x*+3/2, −*y*+3/2, −*z*+1; (iii) −*x*+1/2, −*y*+1, *z*−1/2.
